# Mental comorbidity and multiple sclerosis: validating administrative data to support population-based surveillance

**DOI:** 10.1186/1471-2377-13-16

**Published:** 2013-02-06

**Authors:** Ruth Ann Marrie, John D Fisk, Bo Nancy Yu, Stella Leung, Lawrence Elliott, Patricia Caetano, Sharon Warren, Charity Evans, Christina Wolfson, Lawrence W Svenson, Helen Tremlett, James F Blanchard, Scott B Patten

**Affiliations:** 1 Department of Internal Medicine, University of Manitoba, Winnipeg, Canada; 2 Department of Community Health Sciences, University of Manitoba, Winnipeg, Canada; 3 Health Sciences Centre, GF-543. 820 Sherbrook Street, Winnipeg, MB, Canada; 4 Departments of Psychiatry and Medicine, Dalhousie University, Halifax, Canada; 5 Faculty of Rehabilitation Medicine, University of Alberta, Edmonton, Canada; 6 College of Pharmacy and Nutrition, University of Saskatchewan, Saskatoon, Canada; 7 Department of Epidemiology and Biostatistics and Occupational Health, McGill University, Montreal, Canada; 8 Research Institute of the McGill University Health Centre, Montreal, Canada; 9 Department of Community Health Sciences, University of Calgary, Calgary, Canada; 10 School of Public Health, University of Alberta, Edmonton, Canada; 11 Surveillance and Assessment, Alberta Health, Edmonton, Canada; 12 Department of Medicine (Neurology), University of British Columbia, Vancouver, Canada

**Keywords:** Multiple sclerosis, Administrative data, Validation, Prevalence, Depression, Anxiety, Bipolar disorder, Schizophrenia

## Abstract

**Background:**

While mental comorbidity is considered common in multiple sclerosis (MS), its impact is poorly defined; methods are needed to support studies of mental comorbidity. We validated and applied administrative case definitions for any mental comorbidities in MS.

**Methods:**

Using administrative health data we identified persons with MS and a matched general population cohort. Administrative case definitions for any mental comorbidity, any mood disorder, depression, anxiety, bipolar disorder and schizophrenia were developed and validated against medical records using a a kappa statistic (k). Using these definitions we estimated the prevalence of these comorbidities in the study populations.

**Results:**

Compared to medical records, administrative definitions showed moderate agreement for any mental comorbidity, mood disorders and depression (all k ≥ 0.49), fair agreement for anxiety (k = 0.23) and bipolar disorder (k = 0.30), and near perfect agreement for schizophrenia (k = 1.0). The age-standardized prevalence of all mental comorbidities was higher in the MS than in the general populations: depression (31.7% vs. 20.5%), anxiety (35.6% vs. 29.6%), and bipolar disorder (5.83% vs. 3.45%), except for schizophrenia (0.93% vs. 0.93%).

**Conclusions:**

Administrative data are a valid means of surveillance of mental comorbidity in MS. The prevalence of mental comorbidities, except schizophrenia, is increased in MS compared to the general population.

## Background

Although depression and anxiety are considered common in MS [1,2], population-based prevalence estimates for these conditions are rare. Even fewer prevalence estimates exist for bipolar disorder and schizophrenia in the MS population, and they vary widely [3,4]. The paucity of population-based studies of mental comorbidity may reflect the challenges of conducting such studies. However, such studies are needed given the impact of mental comorbidity in MS, including lower quality of life and reduced adherence to treatment [5,6]; and to minimize the biases from using clinic-based samples.

Studies of mental comorbidity could potentially use one of several data sources including medical records review, self-report, interviews, or administrative data. Administrative data are population-based in publicly funded health systems such as Canada and are cost-effective and accessible [7]. Such data are useful for assessing the burden of disease at the population level, including health services use and costs [8]. Mental comorbidities can be assessed in clinical samples using structured diagnostic interviews such as the Composite International Diagnostic Interview (CIDI) although these are time consuming and depend heavily on recall of past episodes [9]. Administrative data have the advantage that they are recorded during an episode and need not be recalled later. Administrative data, however, are collected for health system management and are often inadequately validated [7,10]. Indeed, few published case definitions for mental comorbidity have been validated, and efforts to develop and validate case definitions for depression have identified poor concordance with the CIDI Short Form [11], and difficulties distinguishing depression from anxiety [12].

We aimed to validate administrative case definitions for several mental comorbidities in MS, and to describe their prevalence among persons with MS versus a matched cohort from the general population. We hypothesized that the prevalence of depression, anxiety, bipolar disorder and schizophrenia would be higher in the MS population than in the general population.

## Methods

### Administrative data

We conducted this study in Manitoba, Canada, using anonymized administrative data obtained from Manitoba Health (MH) which provides health care services for more than 98% of Manitoba residents [13]. Since 1984, MH has maintained computerized records of health services claims, which can be linked using a unique personal health identification number (PHIN) identifying the person who received the service. Physician claims include the PHIN, service date, and three-digit International Classification of Disease (ICD)-9-CM code for one physician-assigned diagnosis. Hospitalization records include the PHIN, admission and discharge dates, and up to 16 discharge diagnoses. Before 2004, diagnoses were listed using five-digit ICD-9-CM codes and since 2004 they have been listed using ICD-10-CA codes. Since 1996, the Drug Programs Information Network captures outpatient prescription drug dispensations including date, drug name, and drug identification number for Manitoba residents, regardless of payer. The population registry is updated when an individual migrates into or out of Manitoba, or dies.

### Study populations and validation cohort

Using data from 1984 to 2006, we identified all Manitobans with MS using a previously validated administrative case definition [14]. We identified up to 5 controls from the general population for each MS case, matched on sex, year of birth and region of residence (postal code), and excluding anyone with an ICD9/10-code for any demyelinating disease as previously described [14]. As described in detail previously, the medical records of 430 persons with MS were reviewed by a trained abstractor using a standardized data collection form [14,15]. Using each participant’s PHIN, these clinical data were linked with the administrative databases.

### Administrative case definitions

We aimed to develop case definitions for depression, anxiety, bipolar disorder and schizophrenia using established approaches [16]. Developing case definitions for mental comorbidity raised challenges. Although hospital claims provide 5-digit ICD codes, physician claims in Manitoba have only three digits, reducing the specificity of coding. For example, at the 3-digit level, the same code (296) describes bipolar I disorder, most recent episodic manic (296.4) and major depressive disorder recurrent episode (296.3). Therefore, we initially created an ‘omnibus’ definition for mental comorbidity to capture persons with any of the mental comorbidities of interest, followed by an ‘any mood or anxiety disorders’ definition which captured depressive disorders, anxiety disorders and bipolar disorder. Finally, we developed case definitions for individual mental comorbidities. We identified ICD-9/10 codes for the individual comorbidities and the combination definitions (Additional file 1: Table S1).

While incorporating prescription claims might improve specificity of the case definitions, many medications used for mental comorbidities are used off-label for other purposes, particularly in MS [17]. To determine which medications to include in our definitions we compiled a list of antidepressants (N06A), anti-anxiolytics (N05B), anti-psychotics (N05A), combination agents (N06C), and mood-stabilizing agents including anticonvulsants (N05AN01, N03AG01, N03AX09, N03AX12) available in Canada based on the Anatomic Therapeutic Chemical Classification System [18]. A multidisciplinary panel comprised of two pharmacists (CE, MM), a psychiatrist (SBP), a neuropsychologist (JDF), an epidemiologist (SW), and a neurologist (RAM) independently reviewed this list and indicated whether each medication was used (i) for each of the mental comorbidities of interest; (ii) other on-label uses including the specific condition; and (iii) off-label uses, especially for MS. To meet our goal of enhancing the specificity of case definitions with prescription claims, we selected medications considered to be moderately specific for the comorbidities of interest (Additional file 1: Table S1), meaning that the medication could not be used off-label for MS, and could not be used on or off-label for conditions other than mental comorbidities unless an ICD code could easily exclude the condition (e.g. epilepsy).

We developed several case definitions for each comorbidity by varying the number of physician, hospital and prescription claims required and the years of data required to classify a person as affected. Using our validation cohort, we compared the classification of mental comorbidity according to the administrative case definitions versus diagnoses based on medical records review for the 1 to 5 year periods ending in fiscal year 2005/06. We report a kappa (κ) statistic for agreement between administrative and medical records data [19], and the 95% confidence interval (CI) based on the normal approximation to the binomial distribution. We interpreted κ as follows: slight (0-0.20), fair (0.21-0.40), moderate (0.41-0.60), substantial (0.61-0.80), and almost perfect agreement (0.81-1.0) [19]. Kappa indicates the proportion of agreement beyond chance and is calculated as (observed agreement – chance agreement) ÷ (1 – chance agreement). Kappa is affected by the prevalence of the condition of interest, however, such that if prevalence is very high or very low, chance agreement is high and kappa is reduced with a maximum value of less than one [20]. Bias refers to the extent to which the raters (i.e. administrative data versus medical records data) disagree on the proportion of positive (affected) cases; greater bias, meaning a greater difference in the proportion (prevalence) of positive rates, is paradoxically associated with higher kappas. Because both prevalence and bias influence the magnitude of kappa, we also calculated the prevalence index, bias index, and the prevalence and bias-adjusted kappa for our preferred case definitions [20]. We estimated that a sample of 400 persons can detect a k of ≥0.60 (substantial agreement) for comorbidities with ≥4% prevalence if the null hypothesis is k = 0.41, α = 0.05, and β = 0.20. Given the anticipated higher k (≥ 0.70) for bipolar disorder and schizophrenia we estimated that our sample would provide adequate precision for estimates of k with prevalences ≥ 3% for these disorders.

We computed sensitivity, specificity, positive predictive value (PPV) and negative predictive value (NPV) for administrative definitions versus the “gold standard” of medical records review to identify whether an algorithm would be vulnerable to over- or under-estimating the prevalence of the comorbidity. Further, we explored the impact of these misclassifications on epidemiologic estimates by generating a range of ‘true’ prevalence estimates, and calculating the expected value of observed prevalence based on the sensitivity and specificity for the case definition of interest [7,21].

### Prevalence

For each comorbidity, we report the prevalence in the MS and matched cohorts. Once a person met the case definition, he or she was considered affected in all subsequent years while alive and resident in Manitoba. We estimated the point prevalence of the comorbidity on October 1, 2005 using mid-year population figures for denominators and also calculated prevalence ratios (PR) by dividing the prevalence in those with MS by that of the control group. To enhance comparability with other study populations, we age-standardized the findings to the 2001 Canadian population, and calculated 95% CIs using the exact binomial distribution. Using Poisson regression we calculated PRs and 95% CIs comparing the MS and general populations adjusting for age group (20-44, 45-59, ≥ 60 years) and sex. Cell sizes ≤ 5 were suppressed.

The University of Manitoba Health Research Ethics Board and the Manitoba Health Information Privacy Committee approved the study and data access. Participants in the validation cohort provided written informed consent. Statistical analyses were performed using SAS V9.2 (SAS Institute Inc., Cary NC).

## Results

The MS population included 4192 persons and the matched cohort included 20,940 persons (71.7% female). In the validation cohort most participants were White (91.6%), women (77.0%), with a mean (standard deviation) age at MS symptom onset of 33.2 (11.1) years [16]. Mental comorbidity was common in the validation cohort, with 29.7% having any mental comorbidity, 29.2% having a mood or anxiety disorder, 27.5% having a depressive disorder, 6.5% having an anxiety disorder, 0.98% having bipolar disorder and 0.49% having schizophrenia.

### Omnibus definition: any mental comorbidity

**Figure 1:**
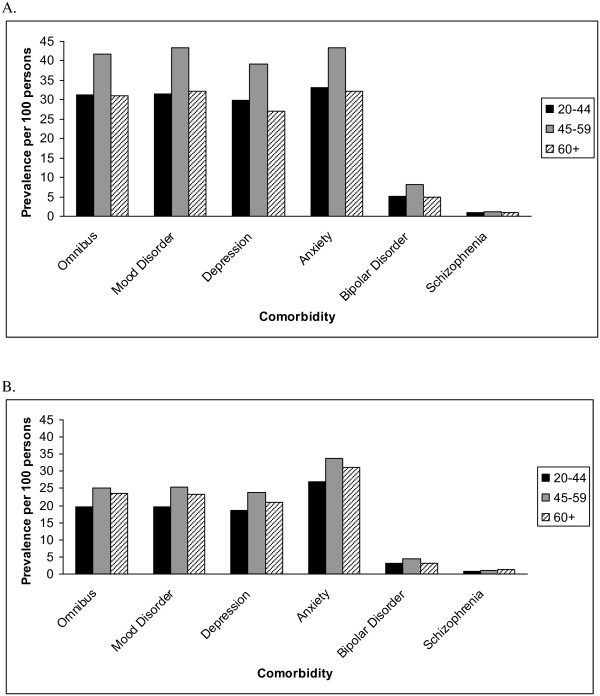
**Age-specific prevalence of mental comorbidity in the MS (A) and general populations (B).** Administrative case definitions used:Omnibus ≥ 1 Hospital or ≥ 5 Physician OR (≥ 1 Physician AND ≥ 4 Prescription claims) in 2 years. Mood disorder ≥ 1 Hospital or ≥ 5 Physician OR (≥ 1 Physician AND ≥ 4 Prescription) in 2 years. Depression ≥ 1 Hospital or ≥ 5 Physician OR (≥ 1 Physician AND ≥ 7 Prescription) in 2 years. Anxiety ≥ 1 Hospital or ≥ 2 Physician OR (≥ 1 Physician AND ≥ 2 Prescription) in 2 years. Bipolar disorder ≥ 1 Hospital or ≥ 3 Physician OR (≥ 1 Physician AND ≥ 3 Prescription) in 5 years. Schizophrenia ≥ 1 Hospital or ≥ 2 Physician in 2 years.

Agreement between the administrative case definitions (labeled A to Z) and medical records ranged from slight to moderate (k = 0.11 to 0.51, Additional file 2: Table S2). The definition with the highest level of agreement required ≥ 1 hospital or ≥ 5 physician claims or ≥ 1 physician claim and ≥ 4 prescription claims in 2 years (definition ‘Q’, k = 0.51); it had a sensitivity of 63% and a specificity of 86.8%. Using definition ‘Q’, the age-standardized prevalence of any mental comorbidity in 2005 was 33.9% (95% CI 32.0-35.9%) in the MS population and 21.9% (95% CI: 21.2-22.6%) in the general population (PR 1.55; 95% CI: 1.36-1.76). In both populations, the peak prevalence occurred in persons aged 45-59 years (Figure 1).

### Any mood or anxiety disorder

Agreement between the case definitions (labeled A to Y) and medical records ranged from slight to moderate (k = 0.10 to 0.50, Additional file 3: Table S3). The highest level of agreement (k = 0.50) was achieved by several similar definitions, including definition ‘O’ (see below); all used prescription claims. Using definition ‘O’, (≥ 1 hospital or ≥ 5 physician or [≥ 1 physician AND ≥ 4 prescription] claims) the age-standardized prevalence of any mood or anxiety disorder in 2005 was 34.8% (95% CI 32.8-36.8%) in the MS population and 22.0% (95% CI: 21.3-22.7%) in the general population (PR 1.58; 95% CI: 1.39-1.80). The similarity of the estimates of any mood or anxiety disorder to those for any mental comorbidity reflects the predominance of mood and anxiety disorders. In both populations, the peak prevalence occurred in persons aged 45-59 years (Figure 1).

### Depressive disorders

Agreement between the case definitions (labeled A to Y, Additional file 4: Table S4) and medical records ranged from slight to moderate (k = 0.11 to 0.49). The highest level of agreement for a definition which did not use prescription claims was moderate (k = 0.44), and used ≥ 1 hospital or ≥4 physician claims in 5 years. Among all case definitions the highest level of agreement (k = 0.49) was achieved by two similar definitions (‘G’ and ‘P’); both used prescription claims. Using definition ‘P’, which required (≥ 1 hospital or ≥ 5 physician claims) or (≥ 1 physician claim and ≥ 7 prescription claims) in 2 years, the age-standardized prevalence of depression in 2005 was 31.7% (95% CI 29.8-33.5%) in the MS population and 20.5% (95% CI: 19.8-21.2%) in the general population (PR 1.60; 95% CI: 1.41-1.82). In both populations, the peak prevalence occurred in persons aged 45-59 years (Figure 1).

### Anxiety disorders

Agreement between the administrative case definitions and medical records ranged from slight to fair (k = 0.02 to 0.23, Additional file 5: Table S5). The highest level of agreement for any definition was fair (definition ‘N’, k = 0.23), and required (≥ 1 hospital or ≥ 2 physician claims) or (≥ 1 physician and ≥ 2 prescription claims) in 2 years. Using definition ‘N’, the age-standardized prevalence of anxiety in 2005 was 35.6% (95% CI 33.7-37.7%) in the MS population and 29.6% (95% CI: 28.8-30.5%) in the general population (PR 1.24; 95% CI: 1.12-1.38). In both populations, the peak prevalence occurred in persons aged 45-59 years (Figure 1).

### Bipolar disorder

Agreement between the administrative case definitions (labeled A to U) and medical records ranged from slight to moderate (k = 0.20 to 0.42, Additional file 6: Table S6). Several definitions had the highest sensitivity of 75% with specificities of 97% or higher. Agreement for these definitions varied slightly, but confidence intervals overlapped. Using definition ‘W’, which required (≥ 1 hospital or ≥ 3 physician claims) or (≥ 1 physician and ≥ 3 prescription claims) in 5 years (k = 0.34), the age-standardized prevalence of bipolar disorder in 2005 was 5.83% (95% CI: 5.01-6.65%) in the MS population and 3.45% (95% CI: 3.17-3.73%) in the general population (PR 1.70; 95% CI: 1.55-1.87). Although the affected number of individuals was small, the prevalence of bipolar disorder was similar across age groups (Figure 1).

### Schizophrenia

Agreement between all of the case definitions (labeled A to O) and medical records ranged from substantial to perfect (k = 0.67 to 1.0, Additional file 7: Table S7). Among case definitions with perfect agreement, the simplest definition with the highest sensitivity (100%) and specificity (99%) required ≥ 1 hospital or ≥ 2 physician claims in 2 years (definition ‘G’). Applying definition ‘G’, the age-standardized prevalence of schizophrenia in 2005 was 0.93% (95% CI: 0.61-1.26%) in the MS population and 0.93% (0.78-1.09%) in the general population (PR 0.95; 95% CI: 0.60-1.51). Although the small number of individuals affected requires cautious interpretation, the prevalence of schizophrenia was similar across age groups (Figure 1).

### Misclassification bias

Table 1 shows the sensitivity, specificity, kappa, prevalence index and bias index for case definitions for which we presented prevalence estimates. The definitions for bipolar disorder, anxiety and schizophrenia have high values for the prevalence index indicating that kappa values will be reduced as compared to populations in which these conditions are more prevalent. Except for anxiety, the bias index was minimal. After adjustment for prevalence and bias, all κ increased except for schizophrenia which was already 1.0. Graphical analysis of misclassification bias suggested that the case definitions perform reasonably well in the expected range of prevalence for mental comorbidity in MS (Additional file 8: Figure S1) [1,2,22-28].

**Table 1:** Impact of prevalence and bias on agreement (kappa) between administrative case definitions and medical records

**Comorbidity**	**Case definition**		**Comparison to medical records**			**Prevalence index**	**Bias index**	**Adjusted kappa**
	**No. years of data**	**No. and type of claims**^**a**^	**Sens (95% CI)**	**Spec (95% CI)**	**Observed kappa (95% CI)**			
Omnibus (Any Mental)	2	≥ 1 H or ≥ 5 P OR (≥ 1 P AND ≥ 4 Rx)	63.5 (54.0, 72.2)	86.8 (82.1, 90.6)	0.51 (0.41, 0.60)	0.42	0.01	0.60
Any Mood or anxiety disorder	2	≥ 1 H or ≥ 5 P OR (≥ 1 P AND ≥ 4 Rx)	62.8 (53.2, 71.7)	86.9 (82.2, 90.6)	0.50 (0.41, 0.60)	0.43	0.01	0.60
Depression	2	≥ 1 H or ≥ 5 P OR (≥ 1 P AND ≥ 7 Rx)	62.2 (52.4, 71.2)	86.7 (82.2, 90.4)	0.49 (0.40, 0.59)	0.45	0	0.60
Bipolar disorder	5	≥ 1 H or ≥ 3P OR (≥ 1 P AND ≥ 3 Rx)	75.0 (19.4, 99.4)	97.5 (95.5, 99.4)	0.30 (0.036, 0.57)	0.95	0.02	0.94
Anxiety	2	≥ 1 H or ≥ 2 P OR (≥ 1 P AND ≥ 2 Rx)	42.3 (23.3, 63.1)	82.2 (78.0, 85.9)	0.23 (0.022, 0.23)	0.74	0.12	0.69
Schizophrenia	2	≥ 1 H or ≥ 2 P	1.0 (15.8, 100)	0.99 (98.6, 100)	1.0 (0, 1.0)	0.99	0	1.0

## Discussion

Few population-based studies have evaluated the prevalence of mental comorbidity in MS [4]. To facilitate such studies, we validated case definitions for mental comorbidities based on hospital, physician and prescription claims. Our case definitions showed almost perfect agreement versus medical records for schizophrenia, and moderate agreement for any mental comorbidity, any mood or anxiety disorder, and depression. The case definition for bipolar disorder had lower agreement, but acceptable sensitivity of 75% and high specificity of 97.5%. The case definition for anxiety had the lowest agreement but a specificity of 82%. Further, persons with MS were at increased risk of depression, anxiety and bipolar disorder, but not schizophrenia when compared to the general population.

Previous validation studies of administrative case definitions for mental comorbidity were often disappointing, and have highlighted the challenges of distinguishing depression from anxiety when using 3-digit ICD codes [11,12]. In a Manitoba study, agreement was only fair (k = 0.26) between surveys and administrative definitions for depression which used hospital, physician and prescription claims [11] although this lower agreement may reflected their use of survey data and a broader range of prescription claims than in our study. Among persons newly treated with antidepressants in Saskatchewan, Canada, agreement between depression identified on physician claims and medical records was moderate (k = 0.54), similar to our findings [29]. We could not identify any published, validated case definitions for anxiety. Thus these validated case definitions augment the ability to conduct population-level surveillance of depression and anxiety. Despite challenges in developing case definitions for depressive and anxiety disorders sensitive and specific definitions were available for bipolar disorder (sensitivity 75%, specificity 97%). Among 225 Americans, inpatient diagnoses of bipolar disorder, outpatient diagnoses of bipolar disorder by mental health providers, and outpatient diagnoses of bipolar disorder by non-mental health providers that were accompanied by a prescription for lithium, carbamazepine or valproate, had false positive rates below 10% [30]. However, we found that bipolar disorder could be identified without such claims. Consistently, administrative case definitions for schizophrenia have performed well, with agreement between hospital claims for schizophrenia and medical records of 93.9-100% [31,32]. In American Medicaid data, the case definition that we validated of either one hospital or two physician claims for schizophrenia in two years identified only 6% false positives (k = 0.76) [33].Collectively, this suggests that administrative data can accurately identify bipolar disorder and schizophrenia in the MS and general populations.

Our approach is informative for researchers wishing to study mental comorbidity in other chronic neurologic diseases, which share the potential problem of under-reporting of comorbidities due to coding biases [34]. While prescription claims may add sensitivity, their use for mental comorbidity is challenging in chronic neurologic diseases because of the frequent off-label use of therapies. By restricting the breadth of prescriptions used, and using them in combination with a physician claim for mental comorbidity we successfully created valid case definitions.

Prior studies suggest that the annual prevalence of depression in MS is up to 14% with a lifetime prevalence of up to 50% [1], and that anxiety disorders affect more than 30% of persons with MS [2,22]. Our crude prevalence estimates of 33% for depression and 37% for anxiety based on two years of administrative data are consistent with those observations. The age-standardized prevalence of bipolar disorder in the MS population was 5.83% (crude prevalence 6.3%), 70% higher than in the general population. Studies in hospital or clinic populations suggested that bipolar disorder affects 0.30% to 13% of the MS population [24-28]. The only one of these studies that used a true general population control group reported that hospitalized persons with MS had bipolar disorder twice as often as hospitalized controls (1.97% vs. 0.92%) [25]. Since that study was limited to hospitalized persons, the prevalence of bipolar disorder may have been underestimated, although the increased risk of bipolar disorder in MS was similar to our findings.

The prevalence of schizophrenia was the same in the MS and general populations (0.93%). Two population-based studies, both using administrative data, evaluated the prevalence of psychosis, not limited to schizophrenia. In Taiwan, psychosis affected 7.5% of the MS population and 2.0% of the general population (odds ratio 4.0) [35]. In Alberta, Canada only 0.8% of MS patients had non-organic pyschoses including schizophrenia-spectrum disorders, and other non-organic psychoses, but this was more than in the general population [4]. Our findings of an absence of an increased prevalence of schizophrenia in our MS population suggest a lack of increased risk which may reflect differences in the psychotic disorders studied (all versus schizophrenia alone), as well as the small number of persons with schizophrenia.

Medical records review for the validation cohort did not involve all records of all health care providers over the lifetime of study participants. For practical reasons we also compared medical records to administrative data for the 1 to 5 year period ending in fiscal year 2005/06, rather than from 1984 onward. Both factors may have reduced agreement between the data sources. Like medical records, administrative data only allow us to identify mental comorbidities for which the patient has been treated; undiagnosed mental comorbidity cannot be captured without a direct patient assessment. This study had several strengths, however. We validated the case definitions in a population similar to the one in which it was applied, the design was population-based, we used matched general population controls, and we used multiple types of administrative data.

## Conclusions

Our findings suggest that administrative data can be used for surveillance for mental comorbidities in MS, and should facilitate studies of the impact of mental comorbidity on health outcomes captured by administrative data such as health care utilization. Our findings also provide population-based data emphasizing the increased prevalence of a range of mood and anxiety disorders in MS.

## Abbreviations

CIDI: Composite International Diagnostic Interview;CI: Confidence interval;ICD: International Classification of Disease;K: Kappa;MH: Manitoba Health;MS: Multiple sclerosis;NPV: Negative predictive value;PHIN: Personal Health Identification Number;PPV: Positive predictive value;PR: Prevalence ratio. CIHR Team in the Epidemiology and Impact of Comorbidity on Multiple Sclerosis includes. Ruth Ann Marrie (University of Manitoba), Bo Nancy Yu (University of Manitoba), Stella Leung (University of Manitoba), Lawrence Elliott (University of Manitoba), Patricia Caetano (University of Manitoba), James F Blanchard (University of Manitoba), Lawrence W. Svenson (University of Alberta), Joanne Profetto-McGrath (University of Alberta), Sharon Warren (University of Alberta), Christina Wolfson (McGill University), Nathalie Jette (University of Calgary), Scott B Patten (University of Calgary), Charity Evans (University of Saskatchewan), Helen Tremlett (University of British Columbia), John Fisk (Dalhousie University), Virender Bhan (Dalhousie University), Michelle Ploughman (Memorial University)

## Competing interests

Ruth Ann Marrie receives research funding from: Canadian Institutes of Health Research, Public Health Agency of Canada, Manitoba Health Research Council, Health Sciences Centre Foundation, Multiple Sclerosis Society of Canada, Multiple Sclerosis Scientific Foundation, Rx & D Health Research Foundation, and has conducted clinical trials funded by Bayer Inc. and Sanofi-Aventis.

John Fisk is the Director of the endMS Atlantic Regional Research and Training Centre which is funded by the Multiple Sclerosis Society of Canada. He receives research funding from the Canadian Institutes of Health Research (CIHR) and in the past has received grants, honoraria and consultation fees from AstraZeneca, Bayer, Biogen-Idec Canada, Heron Evidence Development Limited, Hoffmann-La Roche, MAPI Research Trust, Novartis, Sanofi-Aventis, Serono Canada, and QualityMetric Incorporated.

Nancy Yu receives research support from the Canadian International Development Agency, the Multiple Sclerosis Society of Canada, CIHR, and Manitoba Health and Healthy Living.

Stella Leung reports no disclosures.

Lawrence Elliott receives research support from the Canadian Institutes of Health Research, Health Sciences Centre Foundation, Public Health Agency of Canada, and the Multiple Sclerosis Society of Canada.

Patricia Caetano has worked on a research project funded by Amgen.

Charity Evans reports no disclosures.

Sharon Warren receives research funding from the CIHR, the Canadian Health Services Research Foundation, Alberta Health Services and SSHRC.

Christina Wolfson receives research funding from the Multiple Sclerosis Society of Canada, Canadian Institutes of Health Research, Canada Foundation for Innovation, and Public Health Agency of Canada.

Larry Svenson reports no disclosures.

Helen Tremlett currently receives funding from: the Multiple Sclerosis Society of Canada [Don Paty Career Development Award]; US National MS Society [#RG 4202-A-2 (PI)]; Canadian Institutes of Health Research [MOP: #190898 (PI) and MOP-93646 (PI)]; Michael Smith Foundation for Health Research (Scholar award) and the Canada Research Chair program. She has received speaker honoraria and/or travel expenses to attend conferences from: the Consortium of MS Centres, US National MS Society, Swiss Multiple Sclerosis Society, the University of British Columbia Multiple Sclerosis Research Program, Teva Pharmaceuticals and Bayer Pharmaceutical (honoraria declined) and ECTRIMS. Unless otherwise stated, all speaker honoraria are either donated to an MS charity or to an unrestricted grant for use by her research group.

James Blanchard receives research support from the Multiple Sclerosis Society of Canada, CIHR, Bill & Melinda Gates Foundation, Canadian International Development Agency and the United States Agency for International Development.

Scott Patten was a member of an advisory board for Servier, Canada. He has received honoraria for reviewing investigator-initiated grant applications submitted to Lundbeck and Pfizer and has received speaking honoraria from Teva and Lundbeck. He is an Associate Editor for the Canadian Journal of Psychiatry and a member of the editorial board of Chronic Diseases and Injuries in Canada. He is the recipient of a salary support award (Senior Health Scholar) from Alberta Innovates, Health Solutions and receives research funding from the Canadian Institutes for Health Research, the Institute of Health Economics and the Alberta Collaborative Research Grants Initiative.

## Authors’ contributions

RAM, JDF, SW, SBP, and HT conceived of and designed the study initially. RAM, JDF, LE, PC, CE, SW and SBP reviewed and selected diagnostic codes and pharmacotherapies for algorithm development. RAM, NY and SL analyzed the data. All authors assisted in the interpretation of the data. RAM drafted the manuscript. All authors revised the manuscript and approved the final version for publication.

## Pre-publication history

The pre-publication history for this paper can be accessed here:

http://www.biomedcentral.com/1471-2377/13/16/prepub

## Supplementary Material

Additional file 1: Table S1 Diagnosis and medication codes used to identify comorbidities. Click here for file

Additional file 2: Table S2 *Omnibus Definition*: Administrative Claims Case Definitions as Compared to Medical Records Review. Click here for file

Additional file 3: Table S3 *Mood and Anxiety Disorders*: Administrative Claims Case Definitions as Compared to Medical Records Review. Click here for file

Additional file 4: Table S4 *Depression*: Administrative Claims Case Definitions as Compared to Medical Records Review. Click here for file

Additional file 5: Table S5 *Anxiety Disorders*: Administrative Claims Case Definitions as Compared to Medical Records Review. Click here for file

Additional file 6: Table S6 *Bipolar Disorder*: Administrative Claims Case Definitions as Compared to Medical Records Review. Click here for file

Additional file 7: Table S7 *Schizophrenia*: Administrative Claims Case Definitions as Compared to Medical Records Review. Click here for file

Additional file 8: Figure S1 Assessment of misclassification bias for administrative case definitions for mental comorbidities. Click here for file
