# Supplementary material for: Mental comorbidity and multiple sclerosis: validating administrative data to support population-based surveillance
Source: BMC Neurol. 2013 Feb 6;13:16. doi: 10.1186/1471-2377-13-16 (PMC3599013; doi:10.1186/1471-2377-13-16)
Supplement: Additional file 1: Table S1 — Diagnosis and medication codes used to identify comorbidities. [file 1471-2377-13-16-S1.doc]

**eTable 1.** Diagnosis and medication codes used to identify comorbidities

| **Comorbidity** | **ICD**^a^ **-9 codes** | **ICD**^a^ **-10 codes** | **ATC**^b^ **codes** | **Other Considerations** |
| --- | --- | --- | --- | --- |
| Omnibus | 300.0, 300.2, 296.0, 296.1, 296.04, 296.14, 296.4, 296.44, 296.5, 296.54, 296.6, 296.7, 296.8, 295.x, 296.2, 296.3, 298.0, 300.4 | F40, F41, F20.x, F31, F25, F32, F33, F34 | Antipsychotics, antidepressants, anticonvulsants/ mood stabilizers, anxiolytics including  N05AA02, N05AA03, N05AB02, 05AB03, N05AB04, N05AB06, N05AB08, N05AC01, N05AC02, N05AC03, N05AC04, N05AD01, N05AD08, N05AE04, N05AF01, N05AF04, N05AF05, N05AG01, N05AG02, N05AH01, N05AH02, N05AH03, N05AH04, N05AX08, N05AX12, N05AX13, N06AA01, N06AA02, N06AA04, N06AA11, N06AA12, N06AA17, N06AA21, N06AB03, N06AB04, N06AB05, N06AB06, N06AB08, N06AB10, N06AF03, N06AF04, N06AG02, N06AX06, N06AX11, N06AX16, N06AX21, N06AX23, N05AN01,  N03AF01, N03AG01, N03AX09 | Anticonvulsants used were restricted to lamotrigine, carbamazepine, and valproate which are used as mood stabilizers, after excluding persons with epilepsy.  Antidepressants excluded amitriptyline, nortriptyline and buspirone because of their frequent off-label uses. |
| Mood disorders | 300.0, 300.2, 296.0, 296.1, 296.04, 296.14, 296.4, 296.44, 296.5, 296.5, 296.54, 296.6, 296.7, 296.8, 296.2, 296.3, 298.0, 300.4 | F40, F41, F31, F32, F33, F34 | Antidepressants, anticonvulsants/ mood stabilizers, anxiolytics including  N06AA01, N06AA02, N06AA04, N06AA11, N06AA12, N06AA17, N06AA21, N06AB03, N06AB04, N06AB05, N06AB06  N06AB08, N06AB10, N06AF03, N06AF04, N06AG02, N06AX06, N06AX11, N06AX16, N06AX21, N06AX23, N05AB12, N05AB06,  N05AN01, N03AF01, N03AG01, N03AX09 | Anticonvulsants used were restricted to lamotrigine, carbamazepine, and valproate which are used as mood stabilizers, after excluding persons with epilepsy.  Antidepressants excluded amitriptyline, nortriptyline and buspirone because of their frequent off-label uses. |
| Depression | 296.2, 296.3, 298.0, 300.4 | F32, F33, F34 | Antidepressants including  N06AA01, N06AA02, N06AA04, N06AA11, N06AA12, N06AA17, N06AA21, N06AB03, N06AB04, N06AB05, N06AB06  N06AB08, N06AB10, N06AF03, N06AF04, N06AG02, N06AX06, N06AX11, N06AX16, N06AX21, N06AX23 | Antidepressants excluded amitriptyline, nortriptyline and buspirone because of their frequent off-label uses. |
| Anxiety | 300.0, 300.2 | F40, F41 | Anxiolytics including  N05AB12 (Alprazolam),  N05AB06 (Lorazepam) | None of the antidepressants were considered specific for anxiety. Most benzodiazepines were also considered not to be adequately specific for anxiety disorders. |
| Bipolar disorder | 296.0, 296.1, 296.04, 296.14, 296.4, 296.44, 296.5, 296.44, 296.5, 296.54, 296.6, 296.7, 296.8 | F31 | N05AN01 (lithium),  N03AF01 (carbamazepine),  N03AG01 (valproic acid),  N03AX09 (lamotrigine) | Anticonvulsants used were restricted to lamotrigine, carbamazepine, and valproate which are used as mood stabilizers, after excluding persons with epilepsy. |
| Schizophrenia | 295.x | F20.x, F25 | Antipsychotics including  N05AA02, N05AA03, N05AB02, N05AB03, N05AB04, N05AB06, N05AB08, N05AC01, N05AC02, N05AC03, N05AC04, N05AD01, N05AD08, N05AE04, N05AF01, N05AF04, N05AF05, N05AG01, N05AG02, N05AH01, N05AH02, N05AH03, N05AH04, N05AX08, N05AX12, N05AX13 |  |

a – ICD: International Classification of Disease; b – ATC: Anatomic Therapeutic Chemical System
