# Supplementary material for: Mental comorbidity and multiple sclerosis: validating administrative data to support population-based surveillance
Source: BMC Neurol. 2013 Feb 6;13:16. doi: 10.1186/1471-2377-13-16 (PMC3599013; doi:10.1186/1471-2377-13-16)
Supplement: Additional file 2: Table S2 — Omnibus Definition: Administrative Claims Case Definitions as Compared to Medical Records Review. [file 1471-2377-13-16-S2.doc]

**eTable 2.** *Omnibus* *Definition*: Administrative Claims Case Definitions as Compared to Medical Records Review

| **Name** | **Case Definition** | | **Sens**  **(95% CI)** | **Spec**  **(95% CI)** | **PPV**  **(95% CI)** | **NPV**  **(95% CI)** | **Kappa**  **(95% CI)** |
| --- | --- | --- | --- | --- | --- | --- | --- |
|  | **No. Years of Data** | **No. and type of claims^a^** |  |  |  |  |  |
| A | 1 | ≥1 H or P | 33.9  (25.3, 43.3) | 90.1  (85.9, 93.3) | 59.1  (46.3, 71.0) | 76.3  (71.3, 80.9) | 0.27  (0.17, 0.38) |
| B | 1 | ≥1 H or ≥2P | 23.5  (16.1, 32.3) | 93.7  (90.2, 96.3) | 61.4  (45.5, 75.6) | 74.3  (69.4, 78.9) | 0.21  (0.11, 0.31) |
| C | 1 | ≥1 H or ≥3P | 15.6  (9.55, 23.6) | 96.3  (93.3, 98.2) | 64.3  (44.1, 81.4) | 73.0  (68.1, 77.5) | 0.15  (0.066, 0.24) |
| D | 1 | ≥1 H or ≥5P | 9.57  (4.87, 16.5) | 98.5  (96.3, 99.6) | 73.3  (44.9, 92.2) | 72.0  (67.2, 76.5) | 0.11  (0.035, 0.18) |
| E | 1 | ≥1 H or ≥3P OR (≥1P AND ≥1 Rx) | 30.4  (22.2, 39.7) | 94.4  (91.1, 96.9) | 70.0  (55.4, 82.1) | 76.3  (71.3, 80.7) | 0.30  (0.20, 0.40) |
| F | 1 | ≥1 H or ≥3P OR (≥1P AND ≥2 Rx) | 29.6  (21.4, 38.8) | 94.4  (91.1, 96.9) | 69.4  (54.6, 81.7) | 76.0  (71.1, 80.5) | 0.29  (0.19, 0.39) |
| G | 1 | ≥1 H or ≥3P OR (≥1P AND ≥3 Rx) | 29.6  (21.4, 38.8) | 94.5  (91.1, 96.9) | 69.4  (54.6, 81.7) | 76.0  (71.1, 80.5) | 0.29  (0.19, 0.39) |
| H | 1 | ≥1 H or ≥5P OR (≥1P AND ≥3 Rx) | 27.8  (19.9, 36.9) | 95.2  (92.0, 97.4) | 71.1  (55.7, 83.6) | 75.7  (70.8, 80.2) | 0.28  (0.18, 0.38) |
| I | 1 | ≥1 H or ≥5P OR (≥1P AND ≥4 Rx) | 27.0  (19.1, 36.0) | 95.6  (92.4, 97.7) | 72.1  (56.3, 84.7) | 75.6  (70.7, 80.0) | 0.28  (0.18, 0.37) |
| J | 2 | ≥1 H or P | 69.5  (60.3, 77.8) | 65.1  (59.1, 70.7) | 45.7  (38.2, 53.4) | 83.5  (77.8, 88.2) | 0.30  (0.21, 0.39) |
| K | 2 | ≥1 H or ≥2P | 63.5  (54.0, 72.2) | 77.2  (71.7, 82.0) | 54.1  (45.3, 62.7) | 83.3  (78.2, 87.7) | 0.39  (0.29, 0.48) |
| L | 2 | ≥1 H or ≥3P | 57.4  (47.8, 66.6) | 87.1  (82.5, 90.9) | 65.3  (55.2, 74.5) | 82.9  (78.0, 87.0) | 0.46  (0.36, 0.56) |
| M |  | ≥1 H or ≥7P | 32.2  (23.8, 41.5) | 95.6  (92.4, 97.7) | 75.5  (61.1, 86,6) | 76.9  (72.0, 81.3) | 0.33  (0.23, 0.43) |
| N | 2 | ≥1 H or ≥3P OR (≥1P AND ≥2 Rx) | 66.1  (56.7, 74.7) | 82.0  (76.9, 86.4) | 60.8  (51.7, 69.4) | 85.1  (80.2, 89.2) | 0.47  (0.37, 0.56) |
| O | **2** | ≥1 H or ≥3P OR (≥1P AND ≥3 Rx) | 66.1  (56.7, 74.7) | 82.0  (56.7, 74.6) | 60.8  (51.7, 69.4) | 85.1  (80.2, 89.2) | 0.47  (0.37, 0.56) |
| P | **2** | ≥1 H or ≥5P OR (≥1P AND ≥3 Rx) | 64.3  (54.9, 73.0) | 85.3  (80.5, 89.3) | 64.9  (55.4, 73.6) | 85.0  (80.2, 89.0) | 0.50  (0.40, 0.59) |
| Q | **2** | ≥1 H or ≥5P OR (≥1P AND ≥4 Rx) | 63.5  (54.0, 72.2) | 86.8  (82.1, 90.6) | 67.0  (57.3, 75.7) | 84.9  (80.1, 88.9) | 0.51  (0.41, 0.60) |
| R | **2** | **≥1 H or ≥5P OR (≥1P AND ≥5 Rx)** | **62.6**  **(53.1, 71.4)** | **87.1**  **(82.6, 90.9)** | **67.3**  **(57.5, 76.0)** | **84.6**  **(79.9, 88.6)** | **0.51**  **(0.41, 0.60)** |
| S | 5 | ≥1 H or P | 76.5  (67.7, 83.9) | 54.0  (47.9, 60.1) | 41.3  (34.6, 48.2) | 84.5  (78.2, 89.5) | 0.25  (0.16, 0.33) |
| T | 5 | ≥1 H or ≥3P | 66.1  (56.7, 74.7) | 79.4  (74.1, 84.1) | 57.6  (48.7, 66.1) | 84.7  (79.7, 88.9) | 0.44  (0.34, 0.53) |
| U | 5 | ≥1 H or ≥5P | 57.4  (47.8, 66.6) | 87.5  (83.0, 91.2) | 66.0  (55.8, 75.2) | 82.9  (78.1, 87.1) | 0.47  (0.37, 0.56) |
| V | **5** | **≥1 H or ≥7P** | **47.8**  **(38.4, 57.3)** | **91.9**  **(88.0, 94.9)** | **71.4**  **(60.0, 81.1)** | **80.6**  **(75.8, 84.9)** | **0.44**  **(0.34, 0.54)** |
| W | 5 | ≥1 H or ≥3P OR (≥1P AND ≥3 Rx) | 71.3  (62.1, 79.3) | 76.1  (70.5, 81.0) | 55.8  (47.4, 64.0) | 86.2  (81.2, 90.3) | 0.44  (0.35, 0.53) |
| X | 5 | ≥1 H or ≥5P OR (≥1P AND ≥3 Rx) | 70.4  (61.2, 78.6) | 80.1  (74.9, 84.7) | 60.0  (51.2, 68.3) | 86.5  (81.7, 90.5) | 0.48  (0.39, 0.57) |
| Y | 5 | ≥1 H or ≥5P OR (≥1P AND ≥4 Rx) | 69.6  (60.3, 77.8) | 80.9  (75.7, 85.4) | 60.6  (51.7, 69.0) | 86.3  (81.4, 90.2) | 0.48  (0.39, 0.58) |
| Z | 5 | ≥1 H or ≥5P OR (≥1P AND ≥5 Rx) | 68.7  (59.4, 77.0) | 81.6  (76.5, 86.0) | 61.2  (52.3, 69.7) | 86.0  (81.2, 90.0) | 0.49  (0.39, 0.58) |

a- Hospital (H), Physician (P), or Prescription (DPIN) Claims. Prescription claims data available from 1996 onward.
