# Supplementary material for: Mental comorbidity and multiple sclerosis: validating administrative data to support population-based surveillance
Source: BMC Neurol. 2013 Feb 6;13:16. doi: 10.1186/1471-2377-13-16 (PMC3599013; doi:10.1186/1471-2377-13-16)
Supplement: Additional file 3: Table S3 — Mood and Anxiety Disorders: Administrative Claims Case Definitions as Compared to Medical Records Review. [file 1471-2377-13-16-S3.doc]

**eTable 3.** *Mood and Anxiety Disorders***:** Administrative Claims Case Definitions as Compared to Medical Records Review

| **Name** | **Case Definition** | | **Sensitivity**  **(95% CI)** | **Specificity**  **(95% CI)** | **PPV**  **(95% CI)** | **NPV**  **(95% CI)** | **Kappa**  **(95% CI)** |
| --- | --- | --- | --- | --- | --- | --- | --- |
|  | **No. Years**  **of Data** | **No. and type of claims^a^** |  |  |  |  |  |
| A | 1 | ≥1 H or P | 33.6  (25.0, 43.1) | 90.1  (86.0, 93.4) | 58.5  (45.5, 70.6) | 76.7  (71.7, 81.2) | 0.27  (0.17, 0.37) |
| B | 1 | ≥1 H or ≥2P | 23.0  (15.6, 31.9) | 93.8  (90.2, 96.3) | 60.5  (44.4, 75.0) | 74.7  (69.8, 79.2) | 0.20  (0.11, 0.30) |
| C | 1 | ≥1 H or ≥3P | 15.0  (9.01, 23.0) | 96.4  (93.4, 98.2) | 63.0  (42.4, 80.6) | 73.3  (68.4, 77.8) | 0.15  (0.059, 0.23) |
| D | 1 | ≥1 H or ≥5P | 8.85  (4.32, 15.7) | 98.5  (96.3, 99.6) | 71.4  (41.9, 91.6) | 72.4  (78.3, 87.8) | 0.10  (0.028, 0.17) |
| E | 1 | ≥1 H or ≥3P OR (≥1P AND ≥1 Rx) | 30.0  (21.8, 39.4) | 94.2  (90.7, 96.6) | 68.0  (53.3, 80.5) | 76.6  (71.7, 81.0) | 0.29  (0.19, 0.39) |
| F | 1 | ≥1 H or ≥3P OR (≥1P AND ≥3 Rx) | 29.2  (21.0) | 94.5  (91.1, 96.9) | 68.7  (53.7, 81.3) | 76.4  (71.5, 80.8) | 0.28  (0.18, 0.39) |
| G | 1 | ≥1 H or ≥5P OR (≥1P AND ≥1 Rx) | 29.2  (20.2, 37.6) | 94.9  (92.0, 97.4) | 70.2  (55.7, 83.6) | 76.5  (71.4, 80.7) | 0.29  (0.19, 0.39) |
| H | 1 | ≥1 H or ≥5P OR (≥1P AND ≥3 Rx) | 28.3  (20.2, 37.6) | 95.2  (92.0, 97.4) | 71.1  (55.6, 83.6) | 76.3  (71.4, 80.6) | 0.29  (0.19, 0.39) |
| I | 2 | ≥1 H or P | 69.0  (53.2, 71.7) | 65.3  (71.9, 82.2) | 45.1  (44.5, 62.1) | 83.6  (59.6, 77.4) | 0.30  (0.20, 0.39) |
| J | 2 | ≥1 H or ≥2P | 62.8  (53.2, 71.7) | 77.4  (71.9, 82.2) | 53.4  (44.5, 62.1) | 83.5  (78.3, 87.8) | 0.38  (0.28, 0.48) |
| K | 2 | ≥1 H or ≥3P | 56.6  (47.0, 65.9) | 87.2  (82.7, 90.9) | 64.6  (54.4, 74.0) | 83.0  (78.1, 87.1) | 0.45  (0.36, 0.55) |
| L | 2 | ≥1 H or ≥3P OR (≥1P AND ≥3 Rx) | 65.5  (56.0, 74.2) | 82.5  (77.4, 86.8) | 60.7  (51.4, 69.4) | 85.3  (80.4, 89.3) | 0.47  (0.37, 0.56) |
| M | 2 | ≥1 H or ≥5P OR (≥1P AND ≥1 Rx) | 65.5  (56.0, 74.2) | 81.0  (75.9, 85.5) | 58.7  (49.6, 67.4) | 85.1  (80.1, 89.1) | 0.45  (0.35, 0.54) |
| **N** | 2 | ≥1 H or ≥5P OR (≥1P AND ≥3 Rx) | 63.7  (54.1, 72.6) | 85.8  (81.1, 89.7) | 64.9  (55.2, 73.7) | 85.1  (80.4, 89.1) | 0.50  (0.40, 0.59) |
| O | **2** | **≥1 H or ≥5P OR (≥1P AND ≥4 Rx)** | **62.8**  **(53.2, 71.7)** | **86.9**  **(82.2, 90.6)** | **66.5**  **(56.6, 75.2)** | **85.0**  **(80.3, 89.0)** | **0.50**  **(0.41, 0.60)** |
| **P** | 5 | ≥1 H or P | 76.1  (67.2, 83.6) | 54.0  (47.9, 60.0) | 40.6  (33.9, 47.5) | 84.6  (78.3, 89.6) | 0.24  (0.16, 0.32) |
| Q | 5 | ≥1 H or ≥2P | 69.0  (59.7, 77.4) | 68.6  (62.7, 74.1) | 47.6  (39.7, 55.5) | 84.3  (78.8, 88.8) | 0.33  (0.24, 0.42) |
| R | 5 | ≥1 H or ≥3P | 65.5  (56.0, 74.2) | 79.6  (74.3, 84.2) | 56.9  (48.0, 65.6) | 84.8  (79.8, 89.0) | 0.43  (0.33, 0.53) |
| S | **5** | **≥1 H or ≥5P** | **56.6**  **(47.0, 65.9)** | **87.6**  **(83.1, 91.2)** | **65.3**  **(55.0, 74.6)** | **83.0**  **(78.2, 87.2)** | **0.46**  **(0.36, 0.56)** |
| **T** | 5 | ≥1 H or ≥3P OR (≥1P AND ≥1 Rx) | 71.7  (62.4, 79.7) | 70.4  (64.6, 75.8) | 50.0  (42.0, 57.9) | 85.8  (80.5, 90.1) | 0.37  (0.28, 0.46) |
| U | 5 | ≥1 H or ≥3P OR (≥1P AND ≥2 Rx) | 71.7  (62.4, 79.7) | 75.2  (69.6, 80.2) | 54.4  (46.0, 62.5) | 86.5  (81.5, 90.6) | 0.43  (0.34, 0.52) |
| V | 5 | ≥1 H or ≥3P OR (≥1P AND ≥3 Rx) | 71.7  (62.4, 79.7) | 76.3  (80.8, 81.2) | 55.5  (47.0, 63.7) | 86.7  (81.8, 90.7) | 0.44  (0.35, 0.53) |
| W | 5 | ≥1 H or ≥5P OR (≥1P AND ≥1 Rx) | 71.7  (62.4, 79.7) | 73.0  (67.3, 78.1) | 52.5  (44.1, 60.3) | 86.2  (81.1, 90.4) | 0.40  (0.31, 0.49) |
| X | 5 | ≥1 H or ≥5P OR (≥1P AND ≥3 Rx) | 71.7  (62.4, 79.8) | 80.3  (75.1, 84.8) | 60.0  (51.2, 68.3) | 87.3  (82.5, 91.1) | 0.49  (0.40, 0.58) |
| Y | 5 | ≥1 H or ≥5P OR (≥1P AND ≥4 Rx) | 71.7  (62.4, 79.7) | 80.7  (75.5, 85.2) | 60.4  (51.6, 68.8) | 87.4  (82.6, 91.2) | 0.50  (0.40, 0.59) |

a- Hospital (H), Physician (P), or Prescription (DPIN) Claims. Prescription claims data available from 1996 onward.
