# Supplementary material for: Mental comorbidity and multiple sclerosis: validating administrative data to support population-based surveillance
Source: BMC Neurol. 2013 Feb 6;13:16. doi: 10.1186/1471-2377-13-16 (PMC3599013; doi:10.1186/1471-2377-13-16)
Supplement: Additional file 4: Table S4 — Depression: Administrative Claims Case Definitions as Compared to Medical Records Review. [file 1471-2377-13-16-S4.doc]

**eTable 4.** *Depression*: Administrative Claims Case Definitions as Compared to Medical Records Review

| **Name** | **Case Definition** | | **Sensitivity**  **(95% CI)** | **Specificity**  **(95% CI)** | **PPV**  **(95% CI)** | **NPV**  **(95% CI)** | **Kappa**  **(95% CI)** |
| --- | --- | --- | --- | --- | --- | --- | --- |
|  | **No. Years**  **of Data** | **No. and type of claims^a^** |  |  |  |  |  |
| A | 1 | ≥1 H or P | 34.2  (25.5, 43.8) | 88.7  (84.5, 92.1) | 53.5  (41.3, 65.4) | 78.1  (73.2, 82.4) | 0.26  (0.15, 0.36) |
| B | 1 | ≥1 H or ≥2P | 24.3  (16.7, 33.4) | 92.5  (88.8, 95.2) | 55.1  (40.2, 69.3) | 76.3  (71.6, 80.7) | 0.20  (0.10, 0.30) |
| C | 1 | ≥1 H or ≥3P | 14.4  (8.47, 22.3) | 94.9  (91.7, 97.1) | 51.6  (33.1, 69.8) | 74.5  (69.8, 78.9) | 0.12  (0..031, 0.21) |
| D | 1 | ≥1 H or ≥4P | 11.7  (6.39, 19.2) | 96.6  (93.8, 98.3) | 56.5  (34.5, 76.8) | 74.3  (69.6, 78.6) | 0.11  (0.028, 0.19) |
| E | 1 | ≥1 H or ≥2P OR (≥1 P AND ≥2 Rx) | 31.5  (23.0, 41.0) | 91.5  (87.7, 94.4) | 58.3  (44.9, 70.9) | 77.9  (73.1, 82.2) | 0.27  (0.16, 0.37) |
| F | 1 | ≥1 H or ≥3P OR (≥1 P AND ≥3 Rx) | 30.6  (22.2, 40.1) | 93.5  (90.0, 96.0) | 64.1  (49.8, 76.8) | 78.1  (73.4, 82.3) | 0.29  (0.19, 0.39) |
| G | 1 | ≥1 H or ≥5P OR (≥1 P AND ≥5 Rx) | 27.9  (19.8, 37.2) | 95.2  (92.1, 97.4) | 68.9  (53.3, 81.8) | 77.7  (73.0, 81.9) | 0.28  (0.18, 0.38) |
| H | 2 | ≥1 H or P | 69.4  (59.9, 77.8) | 63.5  (57.7, 69.0) | 41.8  (34.6, 49.3) | 84.5  (79.1, 89.0) | 0.27  (0.18, 0.36) |
| I | 2 | ≥1 H or ≥2P | 64.9  (55.2, 73.7) | 76.1  (70.8, 80.9) | 50.7  (42.2, 59.2) | 85.1  (80.2, 89.2) | 0.38  (0.28, 0.47) |
| J | 2 | ≥1 H or ≥3P | 56.8  (47.0, 66.1) | 85.7  (81.1, 89.5) | 60.0  (50.0, 69.4) | 83.9  (79.3, 87.9) | 0.43  (0.33, 0.53) |
| K | **2** | **≥1 H or ≥4P** | **46.8**  **(37.3, 56.5)** | **90.1**  **(86.1, 93.3)** | **64.2**  **(52.8, 74.5)** | **81.7**  **(77.1, 85.8)** | **0.40**  **(0.30, 0.50)** |
| **L** | **2** | **≥1 H or ≥5P** | **37.8**  **(28.8, 47.5)** | **92.1**  **(88.4, 95.0)** | **64.6**  **(51.8, 76.1)** | **79.6**  **(75.0, 83.8)** | **0.34**  **(0.24, 0.45)** |
| M | 2 | ≥1 H or ≥2P OR (≥1 P AND ≥2 Rx) | 68.4  (59.0, 77.0) | 73.4  (67.9, 78.4) | 49.4  (41.2, 57.5) | 86.0  (81.1, 90.0) | 0.37  (0.28, 0.47) |
| **N** | 2 | ≥1 H or ≥3P OR (≥1 P AND ≥3 Rx) | 66.7  (57.1, 75.3) | 80.5  (75.5, 84.9) | 56.5  (47.6, 65.1) | 86.4  (81.8, 90.3) | 0.45  (0.32, 0.54) |
| **O** | 2 | ≥1 H or ≥5P OR (≥1 P AND ≥5 Rx) | 63.1  (53.4, 72.0) | 86.0  (81.5, 89.8) | 63.1  (53.4, 72.1) | 86.0  (81.5, 89.8) | 0.49  (0.40, 0.58) |
| **P** | **2** | **≥1 H or ≥5P OR (≥1 P AND ≥7 Rx)** | **62.2**  **(52.4, 71.2)** | **86.7**  **(82.2, 90.4)** | **63.9**  **(54.1, 72.9)** | **85.8**  **(81.3, 89.6)** | **0.49**  **(0.40, 0.59)** |
| Q | 5 | ≥1 H or P | 75.7  (66.6, 83.3) | 51.9  (46.0, 57.7) | 37.3  (31.0, 44.0) | 84.9  (78.8, 89.8) | 0.21  (0.13. 0.29) |
| R | 5 | ≥1 H or ≥2P | 70.0  (60.8, 78.6) | 66.9  (61.2, 72.2) | 44.6  (37.1, 52.2) | 85.6  (60.8, 78.6) | 0.32  (0.22, 0.40) |
| S | 5 | ≥1 H or ≥3P | 65.8  (56.1, 745) | 77.8  (72.6, 82.4) | 52.9  (44.2, 61.4) | 85.7  (80.9, 89.7) | 0.40  (0.31, 0.50) |
| T | 5 | ≥1 H or ≥4P | 62.2  (52.5, 71.2) | 82.6  (77.7, 86.7) | 57.5  (48.1, 66.5) | 85.2  (80.5, 89.1) | 0.44  (0.34, 0.53) |
| U | 5 | ≥1 H or ≥5P | 55.8  (46.1, 65.3) | 86.3  (81.9, 90.1) | 60.8  (50.6, 70.3) | 83.8  (79.1, 87.7) | 0.43  (0.33, 0.53) |
| V | 5 | ≥1 H or ≥2P OR (≥1 P AND ≥2 Rx) | 73.0  (63.7, 81.0) | 64.2  (58.4, 69.7) | 43.5  (36.3, 51.0) | 96.2  (80.9, 90.5) | 0.31  (0.22, 0.39) |
| W | 5 | ≥1 H or ≥3P OR (≥1 P AND ≥3 Rx) | 73.0  (63.7, 81.0) | 74.1  (68.6, 79.0) | 51.6  (43.5, 59.6) | 87.8  (83.1, 91.6) | 0.42  (0.33, 0.51) |
| X | 5 | ≥1 H or ≥5P OR (≥1 P AND ≥5 Rx) | 69.4  (59.9, 77.8) | 80.2  (75.1, 84.6) | 57.0  (48.2, 65.5) | 87.4  (82.8, 91.9) | 0.46  (0.37, 0.56) |
| Y | 5 | ≥1 H or ≥5P OR (≥1 P AND ≥7 Rx) | 69.4  (59.9, 77.8) | 80.5  (75.5, 84.9) | 57.5  (48.6, 66.0) | 87.4  (82.8, 91.1) | 0.47  (0.38, 0.56) |

a- Hospital (H), Physician (P), or Prescription (DPIN) Claims. Prescription claims data available from 1996 onward.
