# Supplementary material for: Mental comorbidity and multiple sclerosis: validating administrative data to support population-based surveillance
Source: BMC Neurol. 2013 Feb 6;13:16. doi: 10.1186/1471-2377-13-16 (PMC3599013; doi:10.1186/1471-2377-13-16)
Supplement: Additional file 5: Table S5 — Anxiety Disorders: Administrative Claims Case Definitions as Compared to Medical Records Review. [file 1471-2377-13-16-S5.doc]

**eTable 5**. *Anxiety Disorders*: Administrative Claims Case Definitions as Compared to Medical Records Review

| **Name** | **Case Definition** | | **Sensitivity**  **(95% CI)** | **Specificity**  **(95% CI)** | **PPV**  **(95% CI)** | **NPV**  **(95% CI)** | **Kappa**  **(95% CI)** |
| --- | --- | --- | --- | --- | --- | --- | --- |
|  | **No. Years**  **of Data** | **No. and type of**  **claims^a^** |  |  |  |  |  |
| A | 1 | ≥1 H or P | 23.1  (8.97, 43.6) | 93.6  (90.7, 95.9) | 20.0  (7.71, 38.6) | 94.6  (91.8, 96.7) | 0.15  (0.0059, 0.31) |
| B | 1 | ≥1 H or ≥2P | 15.4  (4.36, 34.9) | 96.0  (93.5, 97.7) | 21.0  (6.05, 45.6) | 94.3  (91.4, 96.4) | 0.13  (-0.025, 0.29) |
| C | 1 | ≥1 H or ≥3P | 3.85  (0.097, 19.6) | 98.7  (96.9, 99.6) | 16.7  (0.42, 64.1) | 93.7  (90.8, 95.9) | 0.039  (-0.076, 0.15) |
| D | 1 | ≥1 H or ≥5P | 3.85  (0.097, 19.6) | 99.5  (98.1, 99.9) | 33.3  (0.84, 90.6) | 93.8  (90.9, 95.9) | 0.056  (-0.067, 0.18) |
| E | 1 | ≥1 H or ≥2P OR (≥1P AND ≥2 Rx) | 15.4  (4.36, 34.9) | 95.7  (93.2, 97.6) | 20.0  (5.73, 43.7) | 94.2  (92.4, 96.4) | 0.12  (-0.028, 0.28) |
| **F** | 1 | ≥1 H or ≥3P OR (≥1P AND ≥3 Rx) | 3.85  (0.097, 19.6) | 97.9  (95.9, 99.1) | 11.1  (0.28, 48.2) | 93.6  (90.8, 95.9) | 0.025  (-0.083, 0.13) |
| G | 1 | ≥1 H or ≥5P OR (≥1P AND ≥5 Rx) | 3.85  (0.097, 19.6) | 98.9  (97.3, 99.7) | 20.0  (0.50, 71.6) | 93.7  (90.9, 95.9) | 0.045  (-0.073, 0.16) |
| H | 1 | ≥1 H or ≥5P OR (≥1P AND ≥7 Rx) | 3.85  (0.097, 19.6) | 98.9  (97.3, 99.7) | 20.0  (0.50, 71.6) | 93.7  (90.9, 95.9) | 0.045  (-0.073, 0.16) |
| I | 2 | ≥1 H or P | 57.7  (36.9, 76.6) | 73.2  (68.4, 77.6) | 12.9  (7.42, 20.4) | 96.2  (93.2, 98.1) | 0.12  (0.035, 0.20) |
| J | 2 | ≥1 H or ≥2P | 38.5  (20.2, 59.4) | 83.8  (79.7, 87.4) | 14.1  (6.97, 24.4) | 95.2  (92.3, 97.2) | 0.12  (0.014, 0.23) |
| **K** | **2** | **≥1 H or ≥3P** | **30.8**  **(14.3, 51.8)** | **90.7**  **(87.3, 93.4)** | **18.6**  **(8.39, 33.4)** | **95.0**  **(92.2, 97.0)** | **0.16**  **(0.027, 0.30)** |
| M | 2 | ≥1 H or ≥5P | 15.4  (4.36, 34.9) | 94.2  (91.3, 96.3) | 15.4  (4.36, 34.9) | 94.2  (91.3,96.3) | 0.10  (-0.043, 0.23) |
| **N** | **2** | **≥1 H or ≥2P OR (≥1P AND ≥2 Rx)** | **42.3**  **(23.3, 63.1)** | **82.2**  **(78.0, 85.9)** | **14.1**  **(7.25, 23.8)** | **95.4**  **(92.5, 97.4)** | **0.23**  **(0.022, 0.23)** |
| O | 2 | ≥1 H or ≥3P OR (≥1P AND ≥3 Rx) | 34.6  (17.2, 55.7) | 88.0  (84.3, 91.1) | 16.7  (7.91, 29.3) | 95.1  (92.3, 97.1) | 0.15  (0.025, 0.28) |
| P | 2 | ≥1 H or ≥5P OR (≥1P AND ≥5 Rx) | 19.2  (6.55, 39.4) | 91.8  (88.5, 94.3) | 13.9  (4.67, 29.5) | 94.3  (91.4, 96.4) | 0.093  (-0.037, 0.22) |
| Q | 2 | ≥1 H or ≥5P OR (≥1P AND ≥7 Rx) | 19.2  (6.55, 39.4) | 92.6  (89.4, 95.0) | 15.1  (5.11, 31.9) | 94.3  (91.4, 96.4) | 0.10  (-0.031, 0.24) |
| R | 5 | ≥1 H or P | 65.4  (44.3, 82.8) | 61.8  (56.7, 66.7) | 10.6  (6.28, 16.4) | 96.3  (93.0, 98.3) | 0.08  (0.018, 0.14) |
| S | 5 | ≥1 H or ≥2P | 50.0  (29.9, 70.1) | 75.6  (70.9, 79.8) | 12.4  (6.76, 20.2) | 95.6  (92.6, 97.6) | 0.11  (0.019, 0.19) |
| T | 5 | ≥1 H or ≥3P | 38.5  (20.2, 59.4) | 84.9  (80.8, 88.3) | 14.9  (7.40, 25.7) | 95.2  (92.4, 97.2) | 0.13  (0.021, 0.25) |
| U | 5 | ≥1 H or ≥5P | 26.9  (11.6, 47.8) | 91.5  (88.2, 94.1) | 17.9  (7.53, 33.5) | 94.8  (92.0, 96.8) | 0.15  (0.010, 0.29) |
| V | 5 | ≥1 H or ≥2P OR (≥1P AND ≥2 Rx) | 57.7  (36.9, 76.6) | 73.7  (69.0, 78.1) | 13.1  (7.55, 20.8) | 96.2  (93.3, 98.1) | 0.12  (0.038, 0.21) |
| W | 5 | ≥1 H or ≥3P OR (≥1P AND ≥3 Rx) | 46.1  (26.6, 66.6) | 82.4  (78.3, 86.2) | 15.4  (8.21, 25.3) | 95.7  (92.9, 97.6) | 0.15  (0.041, 0.25) |
| X | 5 | ≥1 H or ≥5P OR (≥1P AND ≥5 Rx) | 38.5  (20.2, 59.4) | 87.8  (84.1, 90.9) | 17.8  (8.91, 30.4) | 95.4  (92.6, 97.3) | 0.17  (0.044, 0.30) |
| Y | 5 | ≥1 H or ≥5P OR (≥1P AND ≥7 Rx) | 30.8  (14.3, 51.8) | 88.8  (85.2, 91.8) | 16.0  (7.17, 29.1) | 94.9  (92.1, 97.0) | 0.14  (0.0099, 0.26) |

a- Hospital (H), Physician (P), or Prescription (DPIN) Claims. Prescription claims data available from 1996 onward.
