# Supplementary material for: Mental comorbidity and multiple sclerosis: validating administrative data to support population-based surveillance
Source: BMC Neurol. 2013 Feb 6;13:16. doi: 10.1186/1471-2377-13-16 (PMC3599013; doi:10.1186/1471-2377-13-16)
Supplement: Additional file 6: Table S6 — Bipolar Disorder: Administrative Claims Case Definitions as Compared to Medical Records Review. [file 1471-2377-13-16-S6.doc]

**eTable 6**. *Bipolar Disorder*: Administrative Claims Case Definitions as Compared to Medical Records Review

| **Name** | **Case Definition** | | **Sensitivity**  **(95% CI)** | **Specificity**  **(95% CI)** | **PPV**  **(95% CI)** | **NPV**  **(95% CI)** | **Kappa**  **(95% CI)** |
| --- | --- | --- | --- | --- | --- | --- | --- |
|  | **No. Years**  **of Data** | **No. and type of claims^a^** |  |  |  |  |  |
| A | 1 | ≥1 H or P | 50.0  (6.76, 93.2) | 99.0  (97.7, 99.7) | 33.3  (4.33, 77.7) | 99.5  (98.2, 99.9) | 0.39  (0.01, 0.78) |
| B | 1 | ≥1 H or ≥2P | 25.0  (0.63, 80.6) | 99.2  (97.8, 99.8) | 25.0  (0.63, 80.6) | 99.2  (97.8, 99.8) | 0.24  (-0.16, 0.64) |
| C | 1 | ≥1 H or ≥3P | 25.0  (0.63, 80.6) | 99.2  (97.8, 99.8) | 25.0  (0.63, 80.6) | 99.2  (97.8, 99.8) | 0.24  (-0.16, 0.64) |
| D | 1 | ≥1 H or ≥5P | 25.0  (0.63, 80.6) | 99.7  (98.6, 99.9) | 50.0  (1.26, 98.7) | 99.2  (97.8, 99.8) | 0.33  (-0.16, 0.82) |
| E | 1 | ≥1 H or ≥2P OR (≥1P AND ≥2 Rx) | 25.0  (0.63, 80.6) | 99.2  (97.8, 99.8) | 25.0  (0.63, 80.6) | 99.2  (97.8, 99.8) | 0.24  (-0.17, 0.64) |
| F | 1 | ≥1 H or ≥3P OR (≥1P AND ≥3 Rx) | 25.0  (0.63, 80.6) | 99.2  (97.8, 99.8) | 25.0  (0.63, 80.6) | 99.2  (97.8, 99.8) | 0.24  (-0.17, 0.64) |
| G | 1 | ≥1 H or ≥5P OR (≥1P AND ≥5 Rx) | 25.0  (0.63, 80.6) | 99.7  (97.8, 99.8) | 50.0  (1.26, 98.7) | 99.2  (97.8, 99.8) | 0.33  (-0.16, 0.82) |
| H | 1 | ≥1 H or ≥5P OR (≥1P AND ≥7 Rx) | 25.0  (0.63, 80.6) | 99.7  (97.8, 99.8) | 50.0  (1.26, 98.7) | 99.2  (97.8, 99.8) | 0.33  (-0.16, 0.82) |
| I | 2 | ≥1 H or P | 75.0  (19.4, 99.4) | 96.3  (93.9, 97.9) | 16.7  (3.58, 41.4) | 99.7  (98.6, 99.9) | 0.26  (0.019, 0.50) |
| J | 2 | ≥1 H or ≥2P | 50.0  (6.76, 93.2) | 97.3  (95.2, 98.6) | 15.4  (1.92, 45.4) | 99.5  (98.2, 99.9) | 0.22  (-0.43, 0.49) |
| K | 2 | ≥1 H or ≥3P | 50.0  (6.76, 93.2) | 98.0  (96.1, 99.1) | 20.0  (2.52, 55.6) | 99.5  (98.2, 99.9) | 0.27  (-0.035, 0.58) |
| M | 2 | ≥1 H or ≥5P | 25.0  (0.63, 80.6) | 99.0  (97.5, 99.7) | 20.0  (0.51, 71.6) | 99.2  (97.8, 99.8) | 0.21  (-0.15, 0.58) |
| N | 2 | ≥1 H or ≥2P OR (≥1P AND ≥2 Rx) | 75.0  (19.4, 99.4) | 97.5  (95.4, 98.8) | 23.1  (5.04, 53.8) | 99.7  (98.6, 99.9) | 0.34  (0.052, 0.63) |
| O | 2 | ≥1 H or ≥3P OR (≥1P AND ≥3 Rx) | 75.0  (19.4, 99.4) | 98.3  (96.4, 99.3) | 30.0  (6.67, 65.2) | 99.7  (98.6, 99.9) | 0.42  (0.095, 0.75) |
| P | 2 | ≥1 H or ≥5P OR (≥1P AND ≥5 Rx) | 50.0  (6.76, 93.2) | 99.0  (97.5, 99.7) | 33.3  (4.33, 77.7) | 99.5  (98.2, 99.9) | 0.39  (0.0063, 0.78) |
| Q | 2 | ≥1 H or ≥5P OR (≥1P AND ≥7 Rx) | 25.0  (0.63, 80.6) | 99.0  (97.5, 99.7) | 20.0  (0.50, 71.6) | 99.2  (97.8, 99.8) | 0.21  (-0.15, 0.58) |
| R | 5 | ≥1 H or P | 75.0  (19.4, 99.4) | 94.8  (92.1, 96.7) | 12.5  (2.66, 32.4) | 99.7  (98.5, 99.9) | 0.20  (0.003, 0.40) |
| S | 5 | ≥1 H or ≥2P | 75.0  (19.4, 99.4) | 96.0  (93.6, 97.7) | 15.8  (3.38, 39.6) | 99.7  (98.6, 99.9) | 0.25  (0.016, 0.48) |
| **T** | **5** | **≥1 H or ≥3P** | **75.0**  **(19.4, 99.4)** | **97.0**  **(94.8, 98.4)** | **20.0**  **(4.33, 48.1)** | **99.7**  **(99.7, 99.9)** | **0.30**  **(0.036, 0.57)** |
| U | 5 | ≥1 H or ≥5P | 50.0  (6.76, 93.2) | 97.8  (95.8, 99.0) | 18.2  (2.28, 51.8) | 97.3  (98.2, 99.9) | 0.26  (-0.039, 0.55) |
| V | 5 | ≥1 H or ≥2P OR (≥1P AND ≥2 Rx) | 75.0  (19.4, 99.4) | 96.5  (94.2, 98.1) | 17.6  (3.80, 43.4) | 99.7  (98.6, 99.9) | 0.27  (0.024, 0.52) |
| **W** | **5** | **≥1 H or ≥3P OR (≥1P AND ≥3 Rx)** | **75.0**  **(19.4, 99.4)** | **97.5**  **(95.5, 99.4)** | **23.1**  **(5.04, 53.8)** | **99.7**  **(98.6, 99.9)** | **0.34**  **(0.052, 0.63)** |
| X | 5 | ≥1 H or ≥5P OR (≥1P AND ≥5 Rx) | 50.0  (6.76, 93.2) | 98.0  (96.1, 99.1) | 20.0  (2.52, 55.6) | 99.5  (98.2, 99.9) | 0.27  (-0.035, 0.59) |
| Y | 5 | ≥1 H or ≥5P OR (≥1P AND ≥7 Rx) | 50.0  (6.76, 93.2) | 98.0  (96.1, 99.1) | 20.0  (2.52, 55.6) | 99.5  (98.2, 99.9) | 0.27  (-0.035, 0.59) |

a- Hospital (H), Physician (P), or Prescription (DPIN) Claims. Prescription claims data available from 1996 onward.
