# Supplementary material for: Mental comorbidity and multiple sclerosis: validating administrative data to support population-based surveillance
Source: BMC Neurol. 2013 Feb 6;13:16. doi: 10.1186/1471-2377-13-16 (PMC3599013; doi:10.1186/1471-2377-13-16)
Supplement: Additional file 7: Table S7 — Schizophrenia: Administrative Claims Case Definitions as Compared to Medical Records Review. [file 1471-2377-13-16-S7.doc]

**eTable 7**. *Schizophrenia*: Administrative Claims Case Definitions as Compared to Medical Records Review

| **Name** | **Case Definition** | | **Sensitivity**  **(95% CI)** | **Specificity**  **(95% CI)** | **PPV**  **(95% CI)** | **NPV**  **(95% CI)** | **Kappa**  **(95% CI)** |
| --- | --- | --- | --- | --- | --- | --- | --- |
|  | **No. Years**  **of Data** | **No. and type of claims^a^** |  |  |  |  |  |
| A | 1 | ≥1 H or P | 0.50  (1.26, 98.7) | 1.0  (99.1, 100) | 1.0  (2.5, 100) | 0.99  (98.6, 99.9) | 0.67  (0.049, 1.0) |
| B | 1 | ≥1 H or ≥2P | 0.50  (1.26, 98.7) | 1.0  (99.1, 100) | 1.0  (2.5, 100) | 0.99  (98.6, 99.9) | 0.67  (0.049, 1.0) |
| C | 1 | ≥1 H or ≥3P | 0.50  (1.26, 98.7) | 1.0  (99.1, 100) | 1.0  (2.5, 100) | 0.99  (98.6, 99.9) | 0.67  (0.049, 1.0) |
| D | 1 | ≥1 H or ≥5P | 0.50  (1.26, 98.7) | 1.0  (99.1, 100) | 1.0  (2.5, 100) | 0.99  (98.6, 99.9) | 0.67  (0.049, 1.0) |
| E | 1 | ≥1 H OR ≥2P OR (≥1P AND ≥1 Rx) | 0.50  (1.26, 98.7) | 1.0  (99.1, 100) | 1.0  (2.50, 100) | 0.99  (98.6, 99.9) | 0.66  (0.049, 1.0) |
| F | 2 | ≥1 H or P | 1.0  (15.8, 100) | 0.99  (98.6, 100) | 0.67  (9.43, 99.2) | 1.0  (99.1, 100) | 0.80  (0.41, 1.0) |
| G | 2 | ≥1 H or ≥2P | 1.0  (15.8, 100) | 0.99  (98.6, 100) | 0.67  (9.43, 99.2) | 1.0  (99.1, 100) | 1.0  (0, 1.0) |
| H | 2 | ≥1 H or ≥3P | 1.0  (15.8, 100) | 0.99  (98.6, 100) | 0.67  (9.43, 99.2) | 1.0  (99.1, 100) | 1.0  (0, 1.0) |
| I | 2 | ≥1 H or ≥5P | 1.0  (15.8, 100) | 0.99  (98.6, 100) | 0.67  (9.43, 99.2) | 1.0  (99.1, 100) | 1.0  (0, 1.0) |
| J | 2 | ≥1 H OR ≥2P OR (≥1P AND ≥1 Rx) | 1.0  (15.8, 100) | 0.99  (98.6, 100) | 0.67  (9.43, 99.2) | 1.0  (99.1, 100) | 0.80  (0.41, 1.0) |
| K | 5 | ≥1 H or P | 1.0  (15.8, 100) | 0.99  (98.6, 100) | 0.67  (9.43, 99.2) | 1.0  (99.1, 100) | 0.80  (0.41, 1.0) |
| L | 5 | ≥1 H or ≥2P | 1.0  (15.8, 100) | 1.0  (99.1, 100) | 1.0  (15.8, 100) | 1.0  (99.1, 100) | 1.0  (0, 1.0) |
| M | 5 | ≥1 H or ≥3P | 1.0  (15.8, 100) | 1.0  (99.1, 100) | 1.0  (15.8, 100) | 1.0  (99.1, 100) | 1.0  (0, 1.0) |
| N | 5 | ≥1 H or ≥5P | 1.0  (15.8, 100) | 1.0  (99.1, 100) | 1.0  (15.8, 100) | 1.0  (99.1, 100) | 1.0  (0, 1.0) |
| O | 5 | ≥1 H OR ≥2P OR (≥1P AND ≥1 Rx) | 1.0  (15.8, 100) | 0.99  (98.6, 100) | 0.67  (9.43, 99.2) | 1.0  (99.1, 100) | 0.80  (0.41, 1.0) |

a- Hospital (H), Physician (P), or Prescription (DPIN) Claims. Prescription claims data available from 1996 onward.
