# Supplementary material for: Mental comorbidity and multiple sclerosis: validating administrative data to support population-based surveillance
Source: BMC Neurol. 2013 Feb 6;13:16. doi: 10.1186/1471-2377-13-16 (PMC3599013; doi:10.1186/1471-2377-13-16)
Supplement: Additional file 8: Figure S1 — Assessment of misclassification bias for administrative case definitions for mental comorbidities. [file 1471-2377-13-16-S8.doc]

eFigure 1. Assessment of misclassification bias for administrative case definitions for mental comorbidities

A. Omnibus definition (any mental comorbidity)

B. Any Mood or Anxiety Disorder

C. Depressive Disorder

D. Bipolar Disorder

E. Anxiety Disorder

F. Schizophrenia
